# Selections

**Published:** 1892-07

**Authors:** 


### Selections.

### HAND DISINFECTION.

THE complete disinfection of the hands preparatory to obstetric or operative work, particularly abdominal surgery, is so essential that the recent observations of Dr. Howard A. Kelly[1] are deserving of the widest publicity.

The common pus-producing germs, especially the *staphylococcus pyogenes albus*, are found abundantly on the hands and about the nails of everybody. To remove them and render the hands perfectly aseptic is by no means easy. The most certain method, and therefore the one to be followed, seems to be the plan which Dr. Kelly advocates.

Sixty-five experiments were made to test the adequacy of soap and water alone. The hands were thoroughly scrubbed with strong

[1] American Journal of Obstetrics, No. 12, 1891.

brown soap and hot water from ten to twenty-five minutes. In nine instances the hands were found to be aseptic, but in these nine instances the hands had been washed in bichloride of mercury solution the day before.

Immersion of the hands in solution of bichloride of mercury as strong as 1:500 was found to be insufficient to actually destroy the micro-organisms. After immersion in this solution, the epidermis did not yield cultures, but if the hands were subsequently treated with a *sterile* solution of ammonium sulphide, living germs could be obtained from the epidermis. The bichloride inhibited the growth but did not kill the germs, so that when the mercury was precipitated by the ammonium sulphide, the germs immediately commenced to grow.

By similar experiments, lysol and also peroxide of hydrogen were found to be inadequate. The method which yielded the best results, and which in fifty experiments rendered the hands aseptic in forty-four instances, was the use of potassium permanganate and oxalic acid.

This method, as described by Dr. Kelly, is as follows:

"1. Scrubbing the hands, with especial attention to the nails—not more than one millimetre in length—for ten minutes, in water, frequently changed, at about 104° F.

"2. Immersion of the hands in a solution of permanganate of potash, made by adding an excess of the salt to boiling distilled water, until every part of the hands and lower forearms is stained a deep mahogany red or almost black color. They are then transferred at once to a saturated solution of oxalic acid until completely decolorized, and of a healthy pink color. This decolorization is accompanied by a sense of warmth, due to chemical reaction, and a sharp stinging wherever there is any abrasion of the epidermis.

"3. Washing off the oxalic acid in warm sterilized water."—*Journal American Medical Association.*

---

### EUROPHEN.

THIS new iodine product and antiseptic has now been on trial in the hands of the profession for several months. The results obtained by it have been generally favorable. It was designed to replace iodoform, and it was hoped also that it would prove a substitute for the time-honored specific medication in constitutional syphilis.

Europhen is a yellow, resinous powder, with an odor resembling that of saffron. It is insoluble in water and glycerin, but dissolves easily in alcohol, ether, or chloroform. Solutions decompose rapidly. Europhen is five times lighter than iodoform.

Clinically it appears to be a perfect substitute for iodoform. It prevents the development of micro-organisms, and it is soluble in the same menstrua as iodoform. It has the advantage over iodoform that large percentages of it will remain in solution in fixed oils. Furthermore, it is relatively free from odor and non-toxic.—*Atlanta Medical and Surgical Journal.*

---

### TO DEODORIZE IODOFORM.

THE following combination is allowed by the Addendum of the *Netherland Pharmacopœia* to deodorize iodoform: Carbolic acid, one part; oil of peppermint, two parts; iodoform, one hundred and ninety-seven parts.—*New York Medical Journal.*

---

### PEROXIDE OF HYDROGEN AS A DISINFECTANT OF WATER.

DR. ALTEHOEFER, after giving references to the literature of the subject, gives his own researches on the disinfective power of $H_2O_2$ dissolved in water. He finds that the addition of one per one thousand to ordinary drinking-water, to drinking-water containing sewage, or to water containing typhoid bacillus or cholera bacillus, is quite sufficient to destroy the various saprophytic and pathogenic organisms contained under these conditions, if it is obtained perfectly fresh and kept in good condition, and if it is allowed to act for a period of twenty-four hours. It is specially valuable for the disinfection of drinking-water because it does not affect the taste, does not alter the color, and, in the proportion mentioned, is perfectly innocuous. As regards cost, he calculates that sufficient drinking-water—say ten litres—for a family may be sterilized by means of $H_2O_2$ at a cost of about two pence per diem.—*Quarterly Therapeutic Review.*

